# Subclinical hypothyroidism or central hypothyroidism—The danger of thyroid function misinterpretation

**DOI:** 10.1002/ccr3.1694

**Published:** 2018-08-21

**Authors:** Oluwaseun Anyiam, Billy Cheung, Samer Al‐sabbagh

**Affiliations:** ^1^ East and North Hertfordshire NHS Trust Stevenage Hertfordshire UK; ^2^ Present address: Homerton University Hospital London UK

**Keywords:** acute medicine, adrenal insufficiency, empty sella, endocrinology, hypothyroidism

## Abstract

Correct interpretation of thyroid function tests is critical to providing appropriate care to patients with suspected thyroid disease. It is particularly important to distinguish central hypothyroidism from other types due to the potential of concurrent secondary adrenal insufficiency and thus the need for immediate steroid replacement prior to commencing thyroxine.

## 1 INTRODUCTION

Central hypothyroidism (CH) is a rare condition characterized by the presence of low free‐thyroxine (T4) and/or low free‐triiodothyronine (T3) in conjunction with a low or normal thyroid‐stimulating hormone (TSH). It is important to distinguish CH from other forms of hypothyroidism as it is frequently associated with deficiency of other pituitary hormones, particularly adrenocorticotrophic hormone (ACTH).^1^ ACTH deficiency results in secondary adrenal insufficiency, and if this is not treated prior to commencing thyroxine replacement, a life‐threatening adrenal crisis can ensue.^2^

We present the case of an 80‐year‐old man who was inappropriately labeled with the diagnosis of subclinical hypothyroidism due to incorrect interpretation of thyroid function tests. Fortunately, this was noticed during routine preoperative assessment and the potential consequences of misdiagnosis were avoided. This case emphasizes the importance of interpreting thyroid function tests correctly and outlines several learning points for medical physicians who will regularly encounter patients with hypothyroidism in their clinical practice.

## 2 CASE HISTORY

An 80‐year‐old man was reviewed in the ambulatory care clinic following a referral from an anesthetist. His accompanying documentation reported a background of type 2 diabetes, ischemic heart disease, atrial fibrillation, and chronic kidney disease. His list of regular medications comprised of amitriptyline, bisoprolol, gliclazide, quinine, ramipril, simvastatin, temazepam, warfarin, calcium tablets, and co‐codamol.

He was being considered for surgical excision of a suspicious left ureteric lesion, and during his preoperative assessment, he was noted to be anemic and hypotensive, prompting referral. Upon review, he reported a long history of worsening lethargy, weight loss, and occasional episodes of hypoglycemia but denied any headaches or visual disturbance. He also denied dizziness on standing, but his blood pressures revealed a significant postural drop (91/54 mm Hg lying, 70/51 mm Hg standing). His heart rate was stable at 70 beats per minute, ECG showed sinus rhythm, and he did not display any signs suggestive of dehydration.

Baseline investigations revealed a macrocytic anemia with a hemoglobin of 80 g/L and a mean cell volume of 105 fL—reticulocyte, white cell, and platelet counts were normal. Renal function was also at his baseline, and HbA1c was 37 mmol/mol. To investigate the anemia further, thyroid function and hematinics were checked. Hematinic levels were adequate (vitamin B12 541 ng/L, folate 8.18 ng/L, and ferritin 115.1 μg/L); however, he had a profoundly low T4 of 5.6 pmol/L (normal range 10‐19.8 pmol/L) and borderline elevated TSH of 5.63 mIU/L (normal range 0.35‐5.5 mIU/L).

Review of his previous results revealed that his T4 checked a year prior to this was also low (7.6 pmol/L) with a TSH within the normal range (2.5 mIU/L). Moreover, his thyroid function had been checked several times during the previous 2 years demonstrating a similar pattern and he had been given the label of “subclinical hypothyroidism,” for which he was not on any treatment. The acute medicine team appropriately questioned this diagnosis and following discussion with the endocrine outreach team, the correct interpretation of central hypothyroidism was made. At this point, the decision was made to postpone his surgery to enable further investigation and optimization of his clinical state.

**Figure 1:**
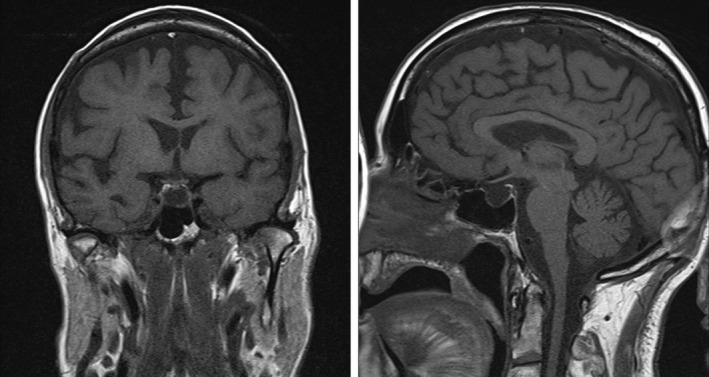
Coronal and sagittal sections of the patient's pituitary magnetic resonance imaging (MRI)

Thyroxine replacement was required; however, prior to this a synacthen stimulation test was arranged which demonstrated an inadequate cortisol response (basal**:** 144 nmol/L, 30 minutes: 333 nmol/L, 60 minutes: 473 nmol/L) and a baseline ACTH level within the normal range. Further pituitary function testing also revealed a slightly elevated prolactin, low insulin‐like growth factor‐1, low luteinizing hormone, and normal follicle‐stimulating hormone (in the context of a low testosterone level). These results were suggestive of pan‐anterior hypopituitarism, and a subsequent MRI pituitary confirmed an almost empty sella with a small area of residual pituitary tissue visible (see Figure 1).

He was commenced on oral hydrocortisone before starting levothyroxine 50 μg once daily. He initially developed palpitations on this dose but gradually settled on a dose of 25 μg twice weekly. By the time of his next review 3 months later, his blood pressure and full blood count had normalized, and he felt much better. He was therefore referred for another urology appointment with a view to rebooking for surgery. However, 2 months later, he was admitted to hospital with a severe respiratory tract infection, and after a seven‐day admission, he unfortunately passed away.

## 3 DISCUSSION

Hypothyroidism is common with a prevalence of approximately 3% in Europe.^3^ It has a wide range of clinical manifestations, the most common being lethargy, cold intolerance, weight gain, and dry skin.^4^ Anemia, caused by reduced stimulation of erythrocyte production,^5^ is also common and can often be the first sign of the condition.^6^, ^7^ It usually resolves with commencement of thyroxine replacement.^8^

Subclinical hypothyroidism is the most common subtype of hypothyroidism and is often described as a state of early mild thyroid failure.^9^ It is diagnosed when TSH is elevated in the presence of a *normal* T4 and T3. It is classified as mild (TSH 4.5‐9 mU/L) or severe (TSH ≥10 mU/L), and thyroxine replacement is generally advised in severe cases or mild cases with symptoms.^10^ In contrast, central hypothyroidism (CH) is thyroid hormone deficiency resulting from insufficient stimulation of a normal thyroid gland.^11^ It is rare, accounting for approximately one per 1000 cases of hypothyroidism.^11^ It is characterized by the presence of a *low* T4 and/or T3 with an associated low or normal TSH. Pituitary mass lesions are by far the most common cause; however, there is a wide range of pituitary disorders that can result in CH.^1^

One such disorder is empty sella syndrome. Empty sella is the term used to describe the herniation of cerebrospinal fluid into the sella turcica causing compression of the pituitary gland and creating the appearance of an “empty” sella turcica on neuroimaging.^12^ It can occur either as an anatomic variant or secondary to a number of pituitary conditions including apoplexy, Sheehan's syndrome, and lymphocytic hypophysitis.^13^ Empty sella syndrome may also develop following treatment of a pituitary adenoma.^13^ Historically, empty sella was thought to be an asymptomatic incidental finding^14^; however, more recent evidence suggests hypopituitarism may occur in up to 60% of cases.^13^ According to current guidance, patients with empty sella syndrome may not necessarily require long‐term follow‐up in an endocrinology clinic.^15^, ^16^ Therefore, such patients could present to general medical physicians with progressive pituitary failure, and knowledge of the potential symptoms (summarized in Table 1) is required to enable timely recognition of this.

**Table 1:** Symptoms of pituitary failure

TSH deficiency	ACTH deficiency	Growth hormone deficiency	Gonadotroph deficiency
Lethargy/fatigue Weakness Cold intolerance Dry skin Weight gain Puffiness Constipation Memory impairment	Lethargy/fatigue Weakness Headache Weight loss/anorexia Nausea/vomiting Hypoglycemia Postural hypotension Impaired mental activity	Reduced energy levels Reduced social activity Reduced muscle mass and strength Reduced bone density Increased body fat Increased cardiovascular risk	Males: loss of libido, erectile dysfunction, reduced muscle mass and bone density, soft testes Females: amenorrhea/oligomenorrhea, hot flushes, breast atrophy, loss of libido, vaginal dryness, reduced bone density

**Figure 2:**
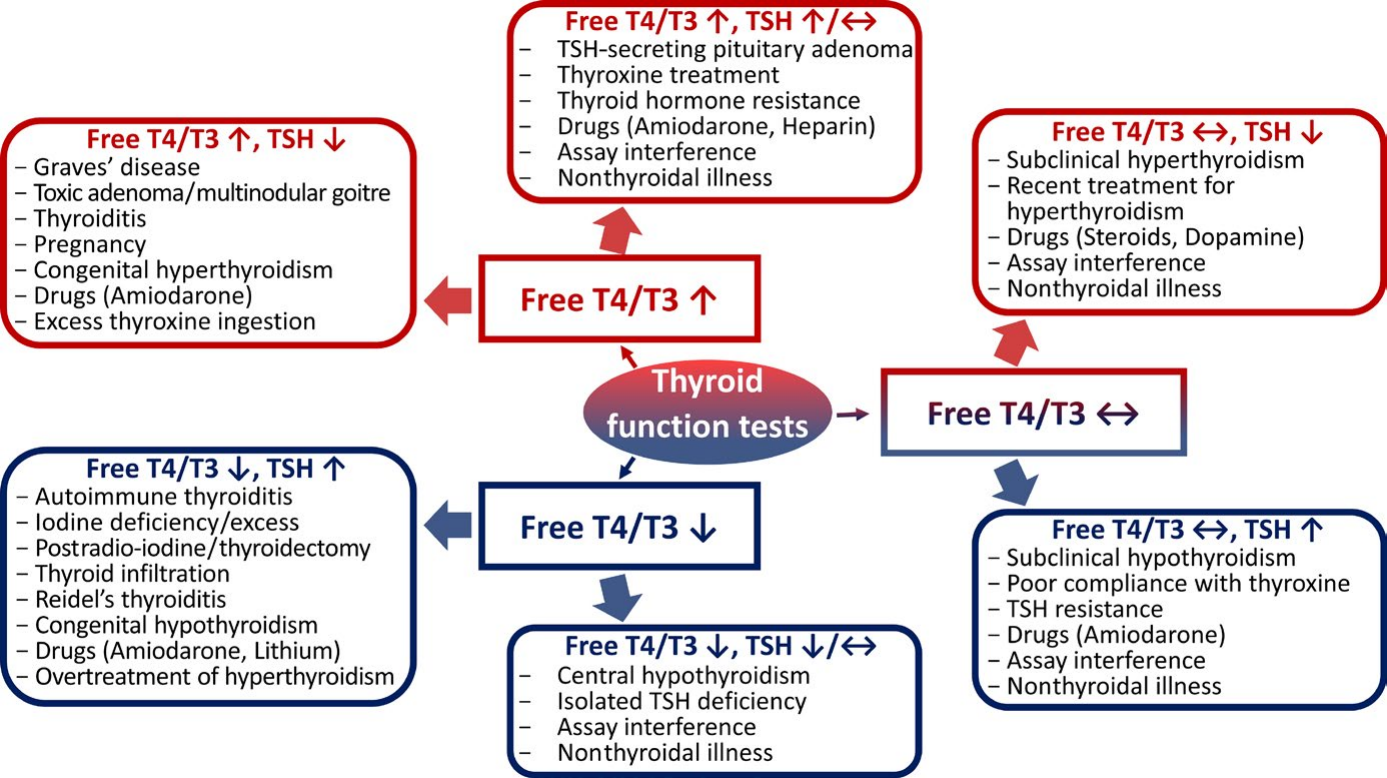
Diagram showing the range of potential thyroid function test results and their causes (adapted from Koulouri and Gurnell, 2013^17^)

To investigate suspected thyroid disease, thyroid function tests are necessary. Results must be accurately interpreted in order to reach the correct diagnosis and institute appropriate management. Figure 2 presents the range of abnormal thyroid function test results and potential associated diagnoses.^17^

Upon diagnosis of hypothyroidism, thyroxine replacement is required. Following institution of thyroxine, patients receive follow‐up with repeat thyroid function tests within 4‐12 weeks and uptitration of doses based on this.^4^ The elderly usually require lower doses of levothyroxine than the general population, and dose titrations are made gradually as overtreatment can be associated with negative effects on health in this group of patients.^4^

In the case of CH, deficiency of other pituitary hormones is highly likely^1^ so a full pituitary screen is imperative. Crucially, concurrent secondary adrenal insufficiency must be identified and treated prior to commencing thyroxine replacement to avoid precipitating an adrenal crisis.^2^, ^11^ Adrenal crises are life‐threatening events with one recent prospective study reporting a mortality rate of approximately 6%.^18^ Adrenal crises have also been described following surgery in patients with undiagnosed adrenal insufficiency.^19^, ^20^ Surgery in this group of patients carries a considerable risk of perioperative morbidity and mortality.^21^ Therefore, it is important to investigate for adrenal insufficiency in any patient with preexisting adrenal or pituitary disease prior to surgery.

The first‐line investigation for adrenal insufficiency is a synacthen stimulation test.^22^ This patient demonstrated an abnormal response to synacthen stimulation, and thus, hydrocortisone replacement was commenced and surgery was delayed. The normal ACTH level in addition to the concomitant deficiency of other pituitary hormones was suggestive of secondary adrenal insufficiency. An insulin tolerance test is considered gold standard to confirm this diagnosis^23^; however, this is unsafe in elderly patients or those with a history of cardiac disease^22^ and so was contraindicated in this case.

It is important to note that it was the presence of anemia which prompted the checking of his thyroid function. Had his thyroid function not been checked in response to this, his underlying diagnosis would not have been established preoperatively. Thyroid disease is an important differential diagnosis for anemia, and thyroid function testing should be considered in patients presenting with this.

Crucial to this case is the fact that this patient had been incorrectly labeled with a diagnosis of subclinical hypothyroidism, despite multiple thyroid function tests demonstrating CH. Diagnosing CH can be challenging, particularly in settings where thyroid function tests consist of TSH measurement in isolation^24^ and another case in which the diagnosis of CH was delayed has been described with serious consequences for the patient concerned.^25^ In our case, the diagnosis and treatment of his hypothyroidism and adrenal insufficiency were delayed for at least 2 years which is likely to have had a significant effect on his quality of life.

A low T4 (or T3) should never be labeled as subclinical hypothyroidism even in the presence of a mildly elevated TSH, which can rarely occur in CH.^4^ Had this diagnosis not been questioned, this patient's true diagnosis would not have been established and he could have undergone surgery in an adrenal insufficient state with potentially devastating consequences. The subsequent institution of appropriate management put him into a more physiologically stable state prior to further consideration for surgery. This also improved his energy levels, mobility, and overall quality of life, even if only for the final 6 months of his life.

## 4 CONCLUSION

The case highlights several learning points for medical physicians. Firstly, this reaffirms the importance of checking thyroid function in patients with anemia. Secondly, correct interpretation of thyroid function tests is of critical importance and this cannot be overstated. If there is any doubt, discussion with an endocrinology specialist is recommended.

Thirdly, in cases of suspected central hypothyroidism, a full pituitary function screen is always necessary to determine deficiency of other pituitary hormones. In particular, adrenal insufficiency must be excluded prior to commencing thyroxine replacement. Adrenal insufficiency must also be detected in preoperative patients as it can lead to severe complications if untreated. Finally, physicians should be aware that patients with empty sella can develop hypopituitarism and should be referred for specialist endocrinology review if they are displaying any suggestive signs or symptoms.

## CONFLICT OF INTEREST

None declared.

## AUTHORSHIP

OA: managed patient and primary manuscript author. BC: managed patient and involved in manuscript writing. SA‐S: managed patient, advised on the discussion, and made critical editions to the manuscript.
